# Free-Energy and Position-Dependent Diffusion Profiles Shape Passive Permeation of a Semisynthetic Naphthoquinone across a Model Lipid Bilayer

**DOI:** 10.1021/acs.jpcb.6c02574

**Published:** 2026-09-04

**Authors:** Fábio J. N. Ferreira, Tallita Marques Machado, Fernanda Guilhon-Simplicio

**Affiliations:** † Faculty of Pharmaceutical Sciences, 67892 Federal University of Amazonas, Manaus, Amazonas 69080-900, Brazil; ‡ Center for Innovation in Artificial Intelligence for Health (CIIA − Saúde/UFMG), Belo Horizonte, Minas Gerais 31270-901, Brazil

## Abstract

Passive membrane permeation depends on both solute partitioning and coordinate-dependent mobility, but their separate and spatial-coupling contributions are rarely quantified within a common resistance framework. CNFD (6b,7-dihydro-5H-cyclopenta­[b]­naphtho­[2,1-*d*]­furan-5,6­(9aH)-dione), a semisynthetic naphthoquinone with low-micromolar cytotoxicity and tumor-growth inhibition in preclinical models, was examined in a four-component model lipid bilayer. Established simulated tempering-enhanced umbrella sampling, WHAM, the Hummer positional-autocorrelation formalism, and the inhomogeneous solubility-diffusion model were combined. Three independent replicas yielded favorable interfacial partitioning (−4.66 kJ mol^–1^) and a positive membrane-center free energy (+12.36 kJ mol^–1^). Controlled profile substitutions showed that PMF heterogeneity and position-dependent diffusion amplified CNFD resistance by factors of 10.54 and 14.89, respectively, whereas their residual spatial-coupling factor was 1.134. The PMF maximum and diffusion minimum were separated by 2.14 nm. The primary intrinsic *P*_eff_ was 9.09 × 10^–2^ cm s^–1^. Applying the same analysis to β-lapachone and plumbagin identified contrasting resistance regimes, with plumbagin combining a favorable center PMF with much lower central resistance localization. These results show how spatially separated free-energy and mobility landscapes govern intrinsic lipid-phase permeation.

## Graphical abstract

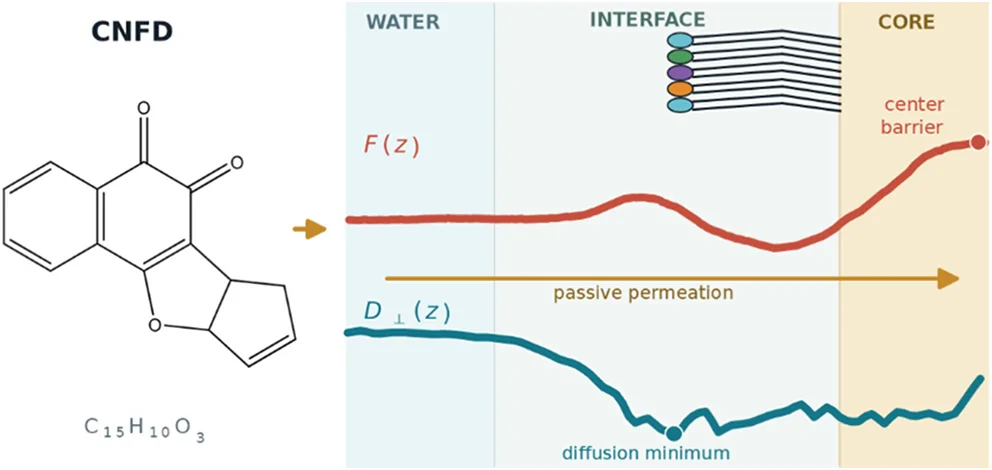

## 1 Introduction

Passive membrane permeability governs the bioavailability, distribution, and efficacy of pharmaceutical compounds and the toxicological profile of environmental xenobiotics.^1,2^ While Lipinski’s rule-of-five provides empirical guidelines,^3^ quantitative prediction of permeability coefficients *P*_eff_ for structurally complex molecules remains challenging due to the interplay of thermodynamic partitioning and kinetic barriers.^4,5^

Quinone-based compounds, particularly naphthoquinones, are ubiquitous in medicinal chemistry as anticancer agents, antimalarials, and mitochondrial electron transport modulators.^6–8^ Despite their therapeutic relevance, the molecular mechanisms underlying their membrane permeation are poorly understood. Traditional experimental methods (Caco-2 assays, PAMPA) provide bulk permeability estimates but lack atomic-level resolution of permeation pathways and energetics.^9,10^

Molecular dynamics (MD) simulations offer mechanistic insights into membrane permeation by explicitly modeling solute-lipid–water interactions.^11,12^ The inhomogeneous solubility-diffusion (ISD) model was introduced by Diamond and Katz,^13^ and was later implemented in atomistic molecular dynamics by Marrink and Berendsen and extended by multiple groups.^14–17^ In this framework, *P*_eff_ is decomposed into two components

eq 1 (ISD Model)

1 $$P_{\mathrm{eff}}^{-1}=\int_{0}^{L}\frac{\exp\left[\beta F\left(z\right)\right]}{D_{⊥}\left(z\right)}dz$$

where *F*(*z*) is the potential of mean force along the membrane normal, *D*_⊥_(*z*) is the position-dependent diffusion coefficient, $\beta =\frac{1}{k_{B}T}$ , and *L* is the integration length. Accurate computation requires (i) converged PMF profiles from enhanced sampling methods and (ii) spatially resolved *D*_⊥_(*z*) from equilibrium or non-equilibrium approaches.^18,19^

Recent advances in enhanced sampling include simulated tempering-enhanced umbrella sampling (ST-eUS), which combines umbrella restraints with a predefined temperature ladder to improve sampling of barrier-crossing configurations. In ST-eUS, the PMF is reconstructed from configurations sampled at the physiological ground temperature.^12^ The position-dependent diffusion coefficient, *D*_⊥_(*z*), was estimated from the restrained-window trajectories using the positional-autocorrelation approach adopted here: the variance of the restrained solute coordinate and its integrated normalized autocorrelation function define the local correlation time and, consequently, *D*_⊥_(*z*).^20^ Together, these analyses provide thermodynamic PMF and kinetic diffusion profiles from a common umbrella-window simulation dataset.

Here, established ST-eUS/WHAM, Hummer positional-autocorrelation, and ISD analyses are used to test a quantitative physical question: how much of the observed membrane resistance arises from PMF heterogeneity, position-dependent diffusion, and their spatial alignment? Controlled profile substitutions provide an exact factorization of these contributions for CNFD. β-Lapachone (LAP) and plumbagin (PLU) are analyzed with the same 46-window, three-replica workflow to determine whether the resulting resistance regime is shared across related naphthoquinones. Published intestinal transport data for LAP and Caco-2 data for PLU provide experimental transport evidence for the comparator compounds, while intrinsic lipid-phase *P*_eff_ remains distinguished from cellular apparent permeability. Together, these analyses quantify how thermodynamic heterogeneity, diffusional heterogeneity, and their spatial coupling define compound-specific resistance regimes.

## 2 Methods

### 2.1 System Preparation

#### 2.1.1 Molecular Structure and Parametrization

CNFD (6b,7-dihydro-5*H*-cyclopenta­[b]­naphtho­[2,1-*d*]­furan-5,6­(9a*H*)-dione; *M*_W_ = 238.24 Da) is a semi-synthetic naphthoquinone derivative with demonstrated in vitro cytotoxic activity against multiple cancer cell lines.^21^ The molecular structure (Figure 1A) was geometry-optimized at the B3LYP/6-311++G­(d,p) level of theory using ORCA 5.0.3.^22^ The neutral 28-atom CNFD topology used CGenFF atom types, partial charges, bonded parameters, and Lennard-Jones parameters, as preserved in the archived production topology. Several analogy-derived bonded terms carried CGenFF penalties above 50 and were not independently reoptimized; force-field transferability is therefore considered among the methodological limitations.^23–25^

**Figure 1:**
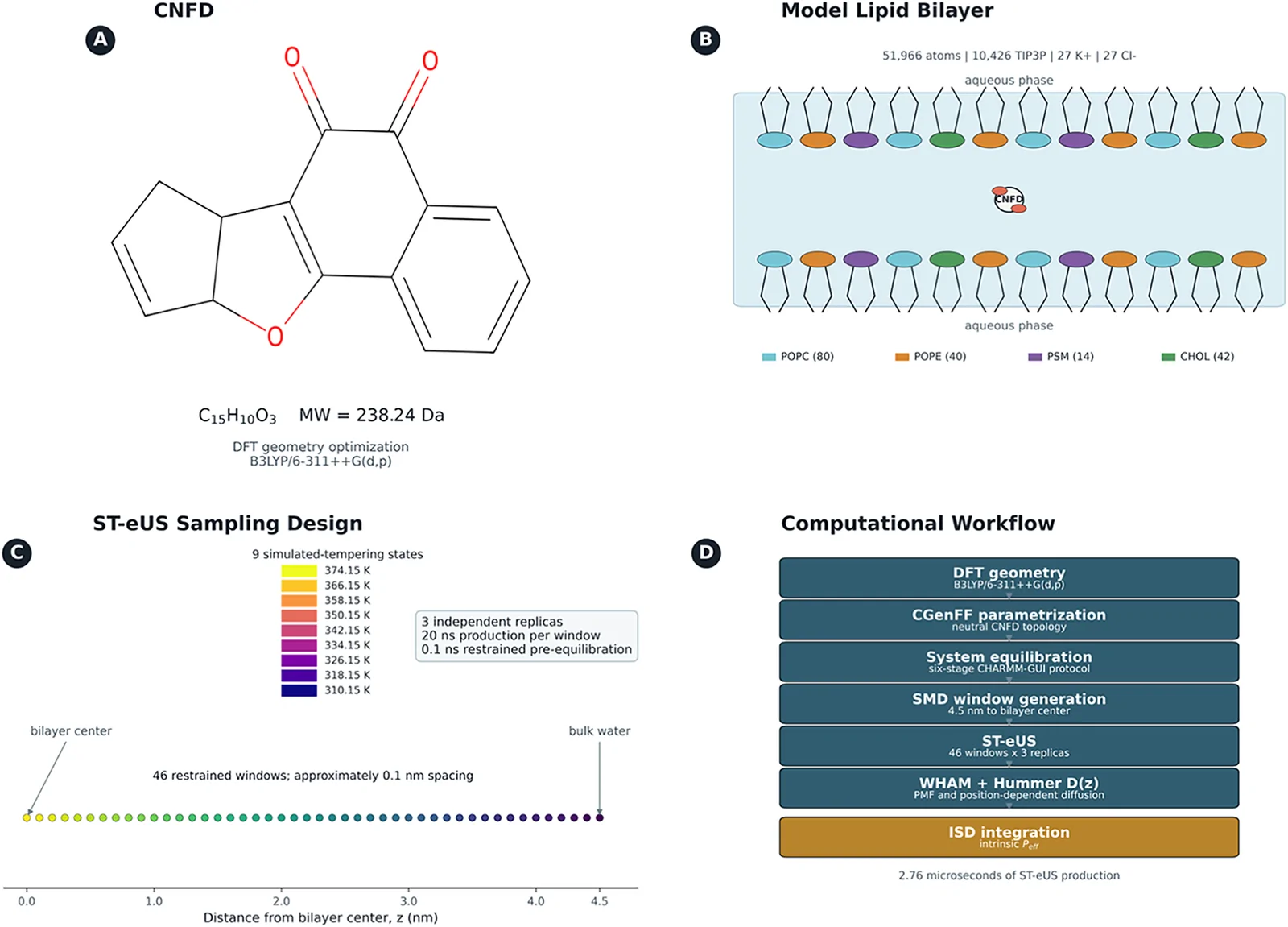
System Setup and Computational Workflow. (A) Two-dimensional chemical structure of CNFD. The fused tricyclic scaffold shows the cyclopentane ring annulated to the naphthofuran system. (B) Schematic representation of the 51,966-atom simulated system containing CNFD, 80 POPC, 40 POPE, 42 cholesterol, 14 PSM, 10,426 TIP3P waters, 27 potassium ions, and 27 chloride ions. (C) Schematic of the ST-eUS protocol: 46 restrained windows from approximately 4.5 nm in bulk water to the bilayer center at approximately 0.1 nm spacing, with nine simulated-tempering states from 310.15 to 374.15 K. (D) Computational pipeline: DFT geometry optimization, CGenFF parametrization, SMD window generation, ST-eUS/WHAM PMF reconstruction, Hummer diffusion analysis, and ISD integration to obtain intrinsic *P*_eff_.

#### 2.1.2 Membrane Construction

A four-component bilayer was constructed with CHARMM-GUI Membrane Builder^26^ (Figure 1B). The archived topology contains 80 POPC, 40 POPE, 42 cholesterol, and 14 PSM molecules (176 lipids total), together with one CNFD, 10,426 TIP3P water molecules, 27 potassium ions, and 27 chloride ions. The resulting neutral periodic system contains 51,966 atoms and provides a defined lipid-phase environment for examining passive CNFD permeation.

#### 2.1.3 Equilibration Protocol

After 5,000 steps of steepest-descent minimization, the system was equilibrated at 310.15 K through a six-stage CHARMM-GUI protocol with progressively released position and dihedral restraints. The first two stages were conducted under NVT conditions for 0.125 ns each. Four subsequent semi-isotropic NPT stages of 0.125, 0.500, 0.500, and 0.500 ns completed the equilibration, with all restraints removed in the final stage. The total post-minimization equilibration time was 1.875 ns.

### 2.2 Steered Molecular Dynamics (SMD)

To generate initial configurations for umbrella sampling, CNFD was pulled from approximately 4.5 nm in bulk water toward the bilayer center using constant-velocity SMD.^27^ The 4.5 ns pull used a rate of 0.001 nm ps-1 and a force constant of 1000 kJ mol-1 nm-2. Forty-six configurations were selected at approximately 0.1 nm intervals from 4.5 to 0.0 nm. Each window then underwent 0.1 ns of restrained equilibration before production.

The center-of-mass coordinate along the membrane normal was selected because CNFD is a compact fused tricyclic scaffold rather than a long flexible solute. The density functional theory (DFT)-optimized structure and subsequent restrained windows did not indicate large-amplitude internal rearrangements that would decouple a single atom or fragment from whole-molecule translocation. Consistent PACF behavior across the 138 umbrella trajectories (mean *R* ^2^ = 0.915) further indicates that the restrained center-of-mass coordinate provided a stable local descriptor of z-dependent fluctuations, while lateral and orientational degrees of freedom remained sampled within each window.

### 2.3 Simulated Tempering-Enhanced Umbrella Sampling (ST-eUS)

#### 2.3.1 Simulation Protocol

Each of the 46 windows accumulated 20 ns of production under NPT conditions with semi-isotropic pressure coupling and a harmonic pull-coordinate force constant of 1000 kJ mol^–1^ nm^–2^ (Figure 1C). Simulated tempering with a temperature ladder of 9 states (310.15, 318.15, 326.15, 334.15, 342.15, 350.15, 358.15, 366.15, 374.15 K; linear spacing, 8 K steps) enhanced sampling of orthogonal degrees of freedom without biasing the reaction coordinate. Temperature exchanges were attempted every 2 ps using the Metropolis criterion.^28^ Three independent replicas with different initial velocities ensured statistical robustness.

#### 2.3.2 PMF Reconstruction

Free energy profiles were reconstructed using the weighted histogram analysis method (WHAM)^29^ implemented in GROMACS 2024.3 *gmx_wham*.^30^ Bootstrapping (*n* = 50) provided uncertainty estimates. PMF curves were zero-referenced to bulk water (z > 3.5 nm) following convention.^31^

#### 2.3.3 Convergence Analysis

Convergence was assessed via (i) inter-replica RMSD, (ii) time-dependent PMF evolution, and (iii) statistical inefficiency (g-factor) analysis. For the RMSD calculation, each replica PMF was independently shifted by its mean value at *z* ≥ 3.5 nm and interpolated onto the common coordinate interval. RMSD was calculated for each of the three unique replica pairs; the reported value is the mean and sample standard deviation of those three pairwise RMSDs. The full common profile was used for the principal comparison, and *z* ≤ 3.5 nm was evaluated separately as a non-bulk sensitivity interval (Figure S2).

### 2.4 Position-Dependent Diffusion within the Hummer Formalism

Position-dependent diffusion coefficients, *D*_⊥_(*z*), were estimated from the restrained ST-eUS trajectories using the positional-autocorrelation relation described by Hummer. For each umbrella window, the first 2 ns of the 20 ns production trajectory were discarded. The normalized positional autocorrelation function, *C*(*t*)/*C*(0), of fluctuations in the restrained center-of-mass coordinate was then evaluated. In the primary analysis, the correlation time was obtained by trapezoidal integration of the normalized autocorrelation function up to its first negative crossing within a maximum lag of 50 ps, and *D*_⊥_(*z*) = ⟨δ*z* ^2^⟩/*τ*_corr_. As an estimator-sensitivity analysis, the PACF was evaluated to 500 ps and its decay over 0 < *t* ≤ 200 ps was fitted to both *C*_fit_(*t*) = exp­(−*t*/*τ*) and *C*_fit_(*t*) = *A*exp (−*t*/*τ*_1_) + (1 – *A*)­exp­(−*t*/*τ*_2_); the fit with the higher *R* ^2^ was retained. The corresponding correlation time was *τ* or *A*τ_1_ + (1 – *A*)*τ*_2_, respectively. The bi-exponential model was retained for 120 of the 138 trajectories, whereas the mono-exponential model was retained for 18 rapidly decorrelating bulk-water trajectories. The fitted-PACF analysis therefore assesses sensitivity to correlation-time treatment within the same Hummer formalism rather than providing an independent diffusion method. A representative fit and its residuals are shown in Figure S5. Because CNFD is a compact fused tricyclic scaffold, its center-of-mass displacement along the membrane normal was used as a practical one-dimensional descriptor of translocation; orientational and lateral motions were not restrained and were evaluated separately as complementary structural observables.

### 2.5 Permeability Calculation and PBC-Aware Structural Profiles

For each compound and diffusion estimator, the sampled half-bilayer resistance was defined as *R*_half_ = ∫_0_ *^L^* exp­[β*F*(*z*)]/*D*_⊥_ *(z)* d*z*, where z = 0 is the bilayer center and L is the bulk-water end point. Bilayer symmetry was then applied explicitly: *R*_full_ = 2*R*_half_ and *P*_eff_ = 1/*R*_full_. The primary result used numerical autocorrelation integration; fitted-PACF integration was retained as an estimator sensitivity.

To separate the thermodynamic and kinetic contributions without new simulations, four profiles were evaluated over the same coordinate interval. The observed resistance, *R*_obs_, retained both *F*(*z*) and *D*_⊥_ *(z)*. The PMF-only resistance, *R*_F_, retained *F*(*z*) and replaced diffusion by *D*_bulk_, defined as the spatial mean for *z* ≥ 3.5 nm. The diffusion-only resistance, *R*_D_, retained *D*_⊥_ *(z)* with *F*(*z*) = 0. The flat reference used *F*(*z*) = 0 and *D*_bulk_, giving *R*_flat_ = L/*D*_bulk_.

The PMF and diffusion amplification factors were *A*_F_ = *R*_F_/*R*_flat_ and *A*_D_ = *R*_D_/*R*_flat_. The residual spatial-coupling factor was *Γ* = *R*_obs_ *R*_flat_/(*R*_F_ *R*_D_), which closes the exact identity *R*_obs_/*R*_flat_ = *A*_F_ *A*_D_ *Γ*. The corresponding RT ln­(A) values express the derived resistance factors on an energy scale; they are decomposition terms rather than independently measured free energies.

For CNFD, LAP, and PLU, PBC-aware structural observables were calculated along the restrained coordinate: water oxygens within 0.35 nm of ligand oxygens, distinct lipid residues within 0.45 nm of the ligand, and the orientation of the ligand principal axis relative to the membrane normal. Membrane and ligand centers were reconstructed by minimum-image coordinates using the umbrella pull-reference atom. Frame values were averaged within each replica and window before compound-level means and SEMs were calculated across the three replicas. Heavy-atom count, radius of gyration, maximum span, and least-squares planarity were additionally evaluated for the optimized solute structures. For PLU, O–H···O geometry was inspected in the optimized structure and three archived SMD configurations to assess geometric plausibility rather than equilibrium occupancy.

For CNFD, the grouped lipid-contact metric was further resolved into POPC, POPE, cholesterol (CHL1), and PSM contributions. Species-specific enrichment was calculated as the fraction of CNFD contacts, defined using a 0.45 nm heavy-atom cutoff, divided by the fraction of locally available lipids within 1.20 nm, after aggregation within each replica and umbrella window. Robustness was assessed using contact cutoffs of 0.40, 0.45, and 0.50 nm and local-availability radii of 1.00, 1.20, and 1.50 nm. Hydrogen bonds were identified using a donor–acceptor distance of at most 0.35 nm and a D–H···A angle of at least 150°. Contact persistence was evaluated at seven representative positions spanning bulk water to the bilayer center, using the native 2 ps trajectory resolution and 0.45 and 0.50 nm thresholds for contact formation and termination, respectively.

### 2.6 Computational Details

Production simulations employed GROMACS 2024.3 with CHARMM36 lipid parameters for the bilayer^32^ CGenFF-compatible parameters for CNFD, and the TIP3P water model.^33^ Electrostatics were treated with particle-mesh Ewald using 1.0 nm real-space electrostatic and van der Waals cutoffs.^34^ Temperature coupling used the velocity-rescale thermostat with *τ*_*t*_ = 1.0 ps,^35^ and semi-isotropic pressure coupling used C-rescale with *τ*_*p*_= 5.0 ps, reference pressure 1.01325 bar, and compressibility 4.5 × 10^–5^ bar^–1^. Bond lengths involving hydrogen were constrained with LINCS,^36^ enabling a 2 fs timestep.

Simulations were performed on the COARACI HPC cluster (University of Campinas – UNICAMP - Brazil). The ST-eUS production dataset comprised 46 windows × 20 ns × 3 replicas = 2,760 ns. Including the 4.5 ns SMD trajectory, 0.1 ns of pre-equilibration for each window and replica (13.8 ns), and the 1.875 ns six-stage system equilibration, the explicitly documented aggregate simulated time was approximately 2.78 μs.

## 3 Results

### 3.1 Interfacial Partitioning and the Central Free-Energy Barrier

The CNFD PMF was reconstructed from the three independent ST-eUS replicas by WHAM for one half of the symmetric bilayer, with the bulk-water region as the free-energy reference. It contains an interfacial minimum of −4.66 kJ mol^–1^ and a positive membrane-center value of +12.36 kJ mol^–1^. CNFD is therefore thermodynamically favored at the interface relative to bulk water, but its transfer into the bilayer center remains unfavorable.

Initial configurations for the 46 restrained windows were selected from the 4.5 ns SMD trajectory. PMFs were subsequently reconstructed from the equilibrated ST-eUS production trajectories, with uncertainty estimated across three independent replicas (Figure 2).

**Figure 2:**
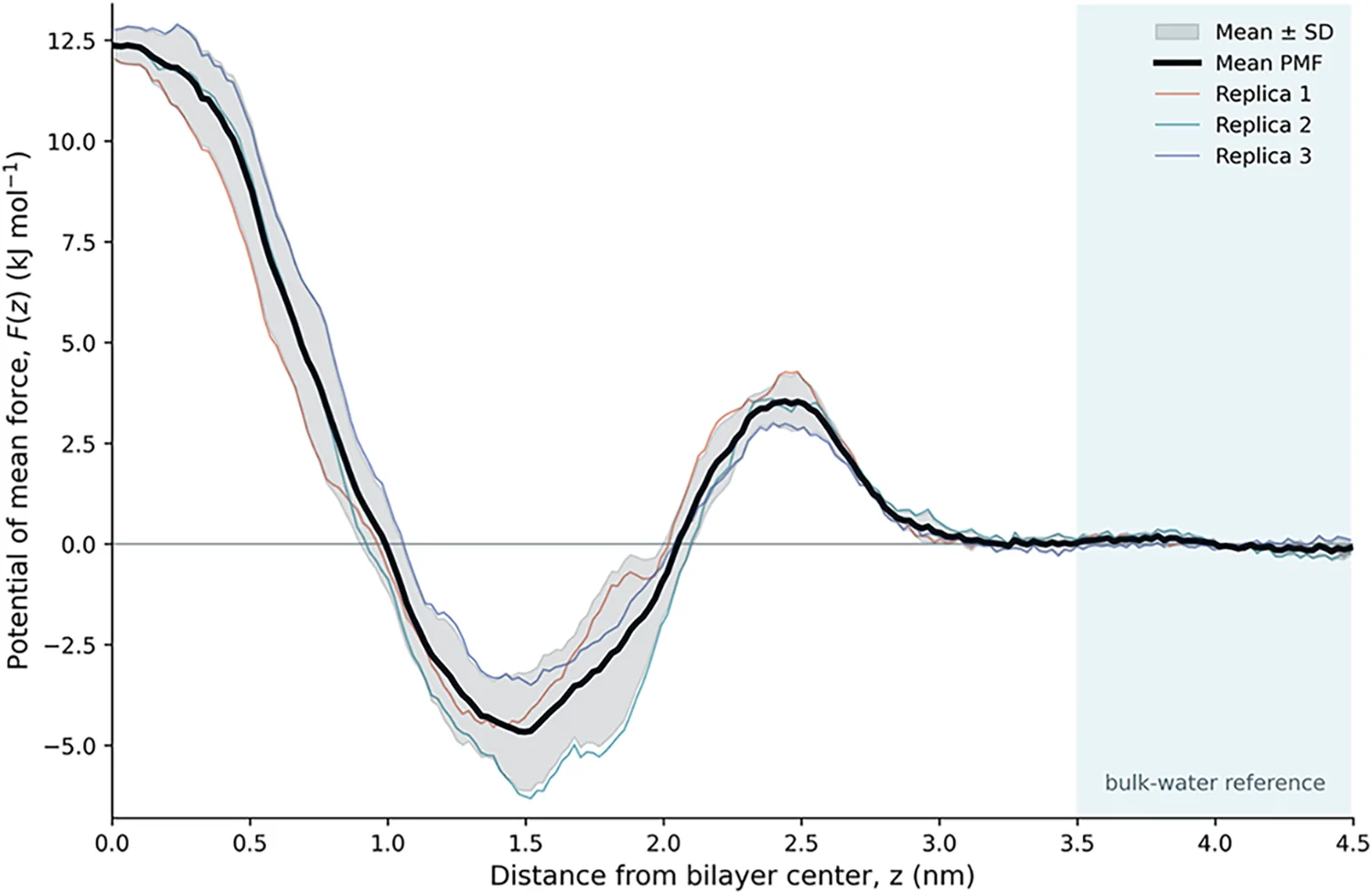
CNFD potential of mean force obtained from ST-eUS/WHAM for one half of the symmetric bilayer, with *z* = 0 at the bilayer center and the bulk-water region (*z* ≥ 3.5 nm) as the reference. Thin colored lines show the three independently normalized replica profiles; the black line is their mean, and the shaded band denotes ±1 SD across replicas. The profile shows an interfacial minimum of −4.66 kJ mol^–1^ and a membrane-center value of +12.36 kJ mol^–1^.

### 3.2 A Distinct Minimum in Position-Dependent Diffusion

The Hummer position-autocorrelation analysis of the 138 restrained production trajectories (46 windows x 3 replicas) showed a strongly position-dependent *D*_⊥_(*z*). The primary numerical-ACF profile spans 57.9-fold when heterogeneity is defined as *D*_max_ /*D*_min_: *D*_min_ = 2.79 × 10^–7^ cm^2^ s^–1^ at *z* = 2.20 nm and *D*_max_ = 1.62 × 10^–5^ cm^2^ s^–1^ at *z* = 4.41 nm. The fitted-PACF sensitivity profile spans 39.8-fold, from 4.51 × 10^–7^ cm^2^ s^–1^ at *z* = 2.32 nm to 1.80 × 10^–5^ cm^2^ s^–1^ at *z* = 4.39 nm. The former is the primary result; the approximately 40-fold value belongs specifically to the fitted-PACF sensitivity analysis.

The PMF maximum and the minimum of *D*_⊥_(*z*) occur at distinct positions along the membrane normal. CNFD encounters its largest free-energy cost at the bilayer center, whereas the lowest local mobility occurs nearer the headgroup-core transition. Both features contribute to the spatial distribution of ISD resistance across the permeation coordinate.

### 3.3 Intrinsic Permeability from ISD Integration

Integration over *z* = 0 to *L* gave the half-bilayer resistance (Figure 3); doubling this value accounted for the second, symmetry-related leaflet before inversion to *P*_eff_. Numerical autocorrelation integration gave a primary CNFD *P*_eff_ of 9.09 × 10^–2^ cm s^–1^, whereas the fitted-PACF sensitivity analysis gave 1.14 × 10^–1^ cm s^–1^. Both estimates use the same PMF and Hummer positional-autocorrelation framework and differ only in correlation-time treatment.

**Figure 3:**
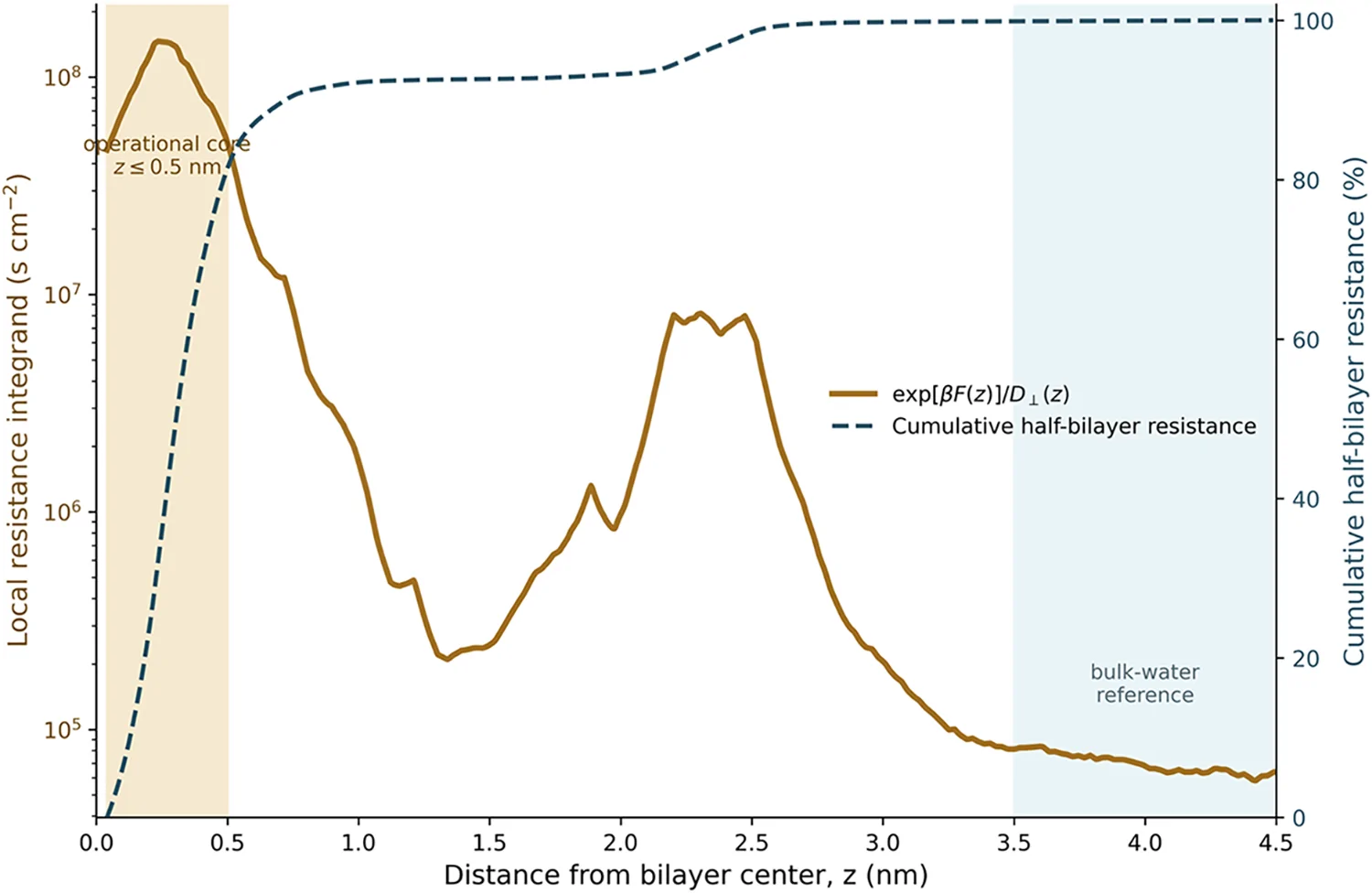
Local ISD resistance analysis for CNFD along the membrane-normal coordinate. The solid curve shows the resistance integrand, exp [*βF*(*z*)]/*D*_⊥_(*z*), derived from the potential of mean force and the position-dependent diffusion coefficient. The dashed curve shows the cumulative fraction of half-bilayer resistance. Shaded regions indicate the operational core (*z* ≤ 0.5 nm) and the bulk-water reference region (*z* ≥ 3.5 nm). Integration over the half-bilayer coordinate, with symmetry accounted for, yields the intrinsic lipid-phase *P*_eff_.

The PMF is highest near the bilayer center, whereas *D*_⊥_(*z*) reaches its minimum nearer the headgroup-core transition. Accordingly, the ISD resistance profile contains a dominant central contribution from the free-energy barrier and an additional contribution from reduced mobility in the headgroup-core region.

Using an operational core defined as *z* ≤ 0.5 nm over the common numerical-ACF integration interval (0.0406 – 4.4888 nm), 81.4% of the integrated half-bilayer resistance lies in the core, which occupies 10.3% of that coordinate interval. Repeating the calculation with each independently bulk-normalized PMF replica while holding the primary *D*_⊥_(*z*) profile fixed gives a descriptive mean of 80.5 ± 2.3% (*n* = 3 PMF replicas); the fitted-PACF sensitivity profile gives 82.8% for the combined PMF. Because the regional fractions depend on the selected core boundary, PMF reference, and correlation-time treatment, they are reported as analysis-specific decompositions of the simulated resistance profile.

### 3.4 Counterfactual Separation of Thermodynamic and Kinetic Resistance

Relative to the flat, zero-PMF/constant-diffusion reference, the CNFD PMF-only profile amplified resistance by *A*_F_ = 10.54, while the diffusion-only profile gave *A*_D_ = 14.89 (Figure 4A). Their residual spatial-coupling factor was *Γ* = 1.134. Expressed as RT ln­(A), these factors correspond to 6.07, 6.97, and 0.33 kJ mol^–1^, respectively. Thus, PMF and diffusional heterogeneity each increase CNFD resistance by approximately one order of magnitude, whereas their additional spatial-alignment contribution is modest. The PMF maximum and diffusion minimum are separated by 2.14 nm, excluding a co-located-extrema interpretation.

**Figure 4:**
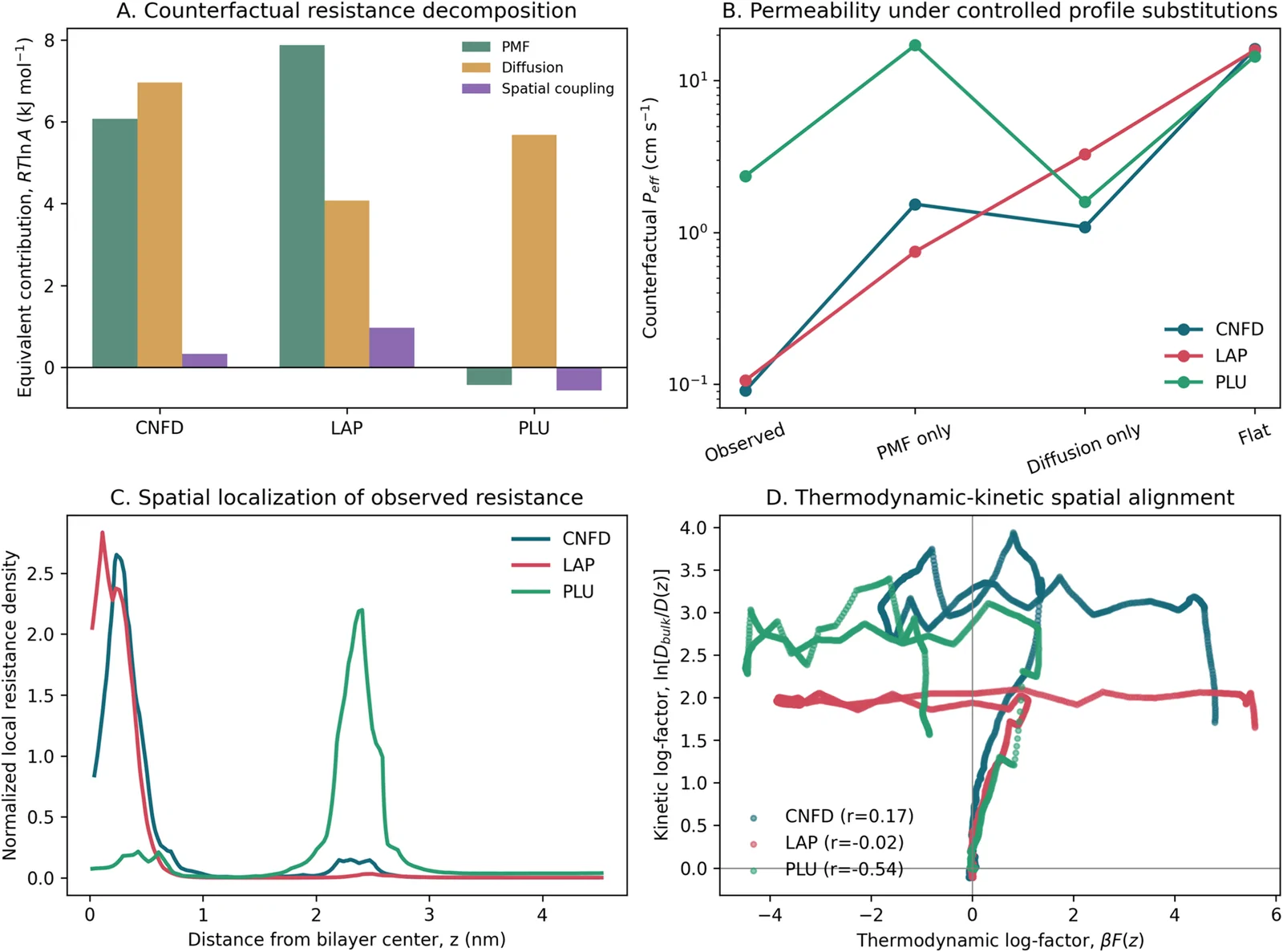
Controlled separation of thermodynamic and kinetic contributions to intrinsic lipid-phase resistance. (A) Equivalent RT ln­(A) contributions from the PMF-only factor, diffusion-only factor, and residual spatial-coupling factor for CNFD, LAP, and PLU. (B) Counterfactual *P*_eff_ values obtained from observed, PMF-only, diffusion-only, and flat profiles. (C) Normalized local resistance densities show the distinct spatial localization of resistance. (D) Coordinate-wise thermodynamic [βF­(*z*)] and kinetic [ln­(*D*_bulk_/*D*_⊥_ *(z)*)] log-factors; *r* denotes their Pearson correlation along the coordinate. All substitutions use the same integration interval and bulk-diffusion definition and are analytical counterfactuals of the sampled profiles, not additional simulations.

The same substitutions expose different physical regimes for the comparators. LAP was more thermodynamically dominated (*A*_F_ = 21.19, *A*_D_ = 4.85, *Γ* = 1.453). PLU instead had a PMF-only factor below unity (*A*_F_ = 0.845), a diffusion factor of 9.07, and *Γ* = 0.800, indicating that its favorable thermodynamic profile partly offsets kinetic resistance. The operational center region (*z* ≤ 0.5 nm) contained 81.4% of CNFD resistance and 94.0% of LAP resistance, but only 6.5% of PLU resistance; the median PLU resistance occurred at *z* = 2.35 nm (Figure 4B–D).

### 3.5 PMF Convergence and Replica Variability

Three independently seeded ST-eUS replicas were simulated, each comprising 46 restrained windows with 20 ns of production per window. For CNFD, the full-profile pairwise PMF RMSDs were 1.260, 1.388, and 1.364 kJ mol^–1^, corresponding to a mean of 1.34 ± 0.07 kJ mol^–1^ (sample SD). Limiting the comparison to the non-bulk interval, *z* ≤ 3.5 nm, gave 1.51 ± 0.08 kJ mol^–1^. These values quantify agreement among the independently sampled ST-eUS profiles; no matched conventional-umbrella-sampling control was included in the present dataset.

The same replica-level workflow was applied to CNFD, LAP, and PLU before compound-level summaries were constructed. Numerical integration of the positional autocorrelation function was used as the primary diffusion estimator, whereas the fitted-PACF analysis was retained to evaluate sensitivity to correlation-time treatment. The reported uncertainty reflects variation across the three simulated replicas; broader model limitations are considered separately below.

## 4 Discussion

### 4.1 Mechanistic Interpretation of CNFD in the Lipid Phase

The controlled substitutions quantify the mechanistic contributions to CNFD resistance. Its PMF and position-dependent diffusion profiles independently amplify resistance by factors of 10.54 and 14.89, while the residual coupling factor of 1.134 adds a comparatively small contribution. Because the PMF maximum and diffusion minimum are 2.14 nm apart, the dominant central resistance does not arise from coincident extrema. Instead, the positive center PMF localizes 81.4% of the integrated resistance within z ≤ 0.5 nm, while reduced mobility elsewhere raises the resistance accumulated across the full coordinate.

Across the PBC-aware profiles, the number of water oxygens within 0.35 nm of the CNFD oxygen atoms decreased as the solute moved from the aqueous/interfacial region toward the bilayer center, whereas the number of neighboring lipid residues increased. This pattern indicates progressive dehydration and increasing membrane association. The local hydration count does not establish persistent cotransport of a particular water molecule or formation of a continuous water defect.

To determine whether the increased lipid contact reflected component-selective association, the aggregate contact metric was resolved into POPC, POPE, cholesterol, and PSM contributions and normalized by the local availability of each lipid species. No component showed enrichment that was reproducible across all three replicas, persisted for at least three consecutive windows, and remained stable across the tested contact and availability cutoffs (Figure S9). Cholesterol showed the largest local enrichment (1.57 ± 0.22 at approximately 0.82 nm), but the enrichment crossed unity in the cutoff-sensitivity analysis. Hydrogen-bond occupancies were low, and median contact durations were similar among the four lipid species. These results indicate that CNFD–lipid contacts primarily reflect the local membrane environment rather than reproducible lipid-specific association.

### 4.2 Methodological Considerations

ST-eUS facilitated sampling within the restrained windows, while SMD generated the initial configurations used to define the umbrella series. The PMF was reconstructed from three independent ST-eUS replicas, and local diffusion was estimated from the corresponding restrained trajectories using a Hummer-derived positional-autocorrelation analysis. Numerical integration of the autocorrelation function served as the primary estimator, whereas fitted-PACF integration was used to assess sensitivity to correlation-time treatment within the same formalism.

For the compact fused CNFD scaffold, center-of-mass displacement along the membrane normal provides a practical one-dimensional descriptor of membrane translocation, while PBC-aware orientation and local-solvation profiles characterize structural changes along that coordinate. Within a harmonically restrained umbrella window, the mean-squared displacement approaches a confinement plateau and therefore does not yield an unrestricted long-time Einstein diffusion coefficient. An unbiased release trajectory initiated at the bilayer center would instead evolve under the PMF gradient while traversing regions with different local diffusivities, so its displacement would combine drift, coordinate-dependent diffusion, and loss of local equilibrium. These observables are therefore not formally equivalent to the local equilibrium *D*_⊥_(*z*). Global Bayesian diffusion-tensor inference, multidimensional reaction coordinates, or transition-based calculations would provide more orthogonal kinetic tests.^37,38^

No empirical viscosity rescaling was applied to the TIP3P-based diffusion coefficients. Consequently, the absolute *D*_⊥_(*z*) and *P*_eff_ values should be interpreted within the present CHARMM/TIP3P model. Experimentally determined octanol/water logP and aqueous hydration free energy values were not available for CNFD, precluding direct comparison with these observables. The bulk-water-referenced PMF and local hydration counts are not equivalent to either measurement. Alternative diffusion estimators and polarizable force fields could further quantify this model dependence.^39^

### 4.3 Comparative Naphthoquinone Resistance Regimes

Applying the same resistance decomposition to all three compounds reveals distinct physical regimes. CNFD combines similarly large PMF and diffusion amplification with weak positive coupling. LAP is more thermodynamically dominated and has stronger positive coupling, whereas PLU has a favorable PMF contribution that partly offsets its diffusion penalty. The observed primary intrinsic *P*_eff_ values were 9.09 × 10^–2^, 1.06 × 10^–1^, and 2.35 cm s^–1^ for CNFD, LAP, and PLU, respectively. CNFD and LAP remain on the same scale within estimator sensitivity, while PLU occupies a distinct low-resistance regime.

PLU also provides a structural contrast for its favorable membrane-center PMF. Relative to CNFD and LAP, PLU has fewer heavy atoms (14 versus 18), a smaller radius of gyration (0.244 versus 0.284–0.286 nm), a shorter maximum span (0.663 versus 0.769–0.770 nm), and an essentially planar optimized scaffold. Near the bilayer center it is almost fully dehydrated and has the largest lipid-contact count after normalization by heavy-atom number (0.722 versus 0.627–0.636). These observations are consistent with compact packing of PLU in the hydrocarbon region and reduced solvent exposure of its polar groups.

A short intramolecular O–H···O geometry was present in the optimized PLU structure and in three archived pulling-path configurations. Similar intramolecular hydrogen-bonding chemistry has been reported for α-phenolic naphthoquinones, including plumbagin.45 These limited configurations support the geometric plausibility of intramolecular hydrogen bonding but are insufficient to quantify its equilibrium occupancy or assign it as the unique origin of the negative center PMF. The structural descriptors and sensitivity analyses are provided in the Supporting Information.

Across the three compounds, thermodynamic and kinetic heterogeneity generate distinct resistance landscapes: CNFD shows comparable amplification by both terms with modest coupling, LAP is thermodynamically dominated, and PLU combines favorable free energy with a diffusion penalty concentrated nearer the membrane interface.

### 4.4 Relationship to Experimental Transport

The calculations and transport experiments address connected but nonidentical parts of membrane passage. If passive, unfacilitated bilayer permeation contributes materially to cellular or intestinal transport, the intrinsic lipid resistance calculated here is one contribution to the total resistance. Caco-2 and in situ measurements additionally contain aqueous boundary layers, cellular architecture, transporters, metabolism, and assay-specific conditions. Accordingly, simulated lipid-phase *P*_eff_ and experimental transport coefficients are treated as complementary observables rather than linked through a universal numerical conversion.

Experimentally, LAP and PLU cross intestinal or cellular barriers at measurable rates (Table 1), establishing that related naphthoquinones can traverse these more complex systems. Within the shared bilayer model, CNFD and LAP have intrinsic *P*_eff_ values on the same scale, whereas PLU has substantially lower intrinsic resistance. Under the conditional hypothesis that direct bilayer permeation is an important route and that nonlipid contributions are comparable, the simulations predict CNFD transport behavior closer to LAP than to PLU. Existing measurements do not test this rank order because the assays are not matched and no direct CNFD Caco-2, PAMPA, or intestinal-perfusion value is available. Separate β-lapachone pharmacokinetic and metabolic studies further show why permeability alone does not determine systemic exposure. A matched experimental series would provide the direct test.^41,42,45^

**Table 1:** Comparative Transport Data for CNFD and Related Naphthoquinones^a^

compound	simulated intrinsic lipid-phase *P*_eff_ (cm s^–1^)	published experimental transport evidence	interpretation
CNFD	9.09 × 10^–2^	no direct Caco-2, PAMPA, or intestinal-perfusion measurement is currently available.	the simulation defines intrinsic permeation through the model lipid phase.
β-Lapachone (LAP)	1.06 × 10^–1^	pure-drug rat small-intestinal *P*_eff_ = (1.17 ± 0.17) × 10^–4^ cm s^–1^.^40^ A formulation study reported Caco-2 *P*_app_ = 2.63 × 10^–5^ cm s^–1^.^43^	intestinal and cellular transport provide external disposition context for a structurally related naphthoquinone.
Plumbagin (PLU)	2.35	Caco-2 A-to-B *P*_app_ = 10.29–15.96 × 10^–6^ cm s^–1^; efflux ratio, 0.57–0.73.^44^	the cellular data complement the favorable central PMF and higher intrinsic lipid-phase permeability observed in the simulations.

a Simulated values are numerical-ACF/Hummer estimates of intrinsic lipid-phase *P*_eff_. Experimental values retain the transport definition and units reported for each assay.

## 5 Conclusions

This work uses established ST-eUS/WHAM, Hummer positional-autocorrelation, and ISD analyses to resolve the physical origins of CNFD resistance in a model lipid bilayer. Controlled profile substitutions showed that PMF heterogeneity and position-dependent diffusion amplify resistance by factors of 10.54 and 14.89, respectively, whereas their residual spatial-coupling factor is only 1.134. Because the PMF maximum and diffusion minimum are separated by 2.14 nm, the central resistance is not a co-located thermodynamic–kinetic bottleneck. The primary intrinsic lipid-phase *P*_eff_ is 9.09 × 10^–2^ cm s^–1^, conditional on the present CHARMM/TIP3P model and numerical-ACF estimator.

The same decomposition distinguishes three naphthoquinone regimes: CNFD has substantial and comparable thermodynamic and kinetic amplification, LAP is more thermodynamically dominated, and PLU combines favorable free energy with a diffusion penalty shifted away from the bilayer center. PLU’s compact, planar, dehydrated, and contact-rich center configuration is consistent with its favorable central PMF, although the present data do not assign a unique causal interaction. Published LAP and PLU transport measurements make the conditional experimental implication explicit: if passive bilayer permeation is an important common route, CNFD is predicted to behave more like LAP than PLU. A matched CNFD/LAP/PLU transport experiment is required to test that prediction.

## Supplementary Material



## Data Availability

All data supporting the findings of this study are available within the article, its Supporting Information, and at the public repository: https://github.com/prifabiojorge/permeability-calculations-using-the-ISD-model (DOI: 10.5281/zenodo.21344010). The repository contains the archived CNFD topology and molecular structures, representative GROMACS parameter files, PMF profiles from all three replicas, the primary numerical-ACF and fitted-PACF diffusion profiles, harmonized comparator profiles and structural summaries, deterministic scripts for ISD integration and metric-validation scripts, and the Python dependencies (requirements.txt). MD trajectories are available from the corresponding author upon reasonable request due to file size constraints. No proprietary or restricted data were used in this study.

## Notes

SMD pulling trajectory; ST-eUS convergence analysis; position-dependent diffusion profile; PMF bootstrap analysis; intrinsic *P*_eff_ sensitivity analysis with a representative parametric PACF fit and residual diagnostics; PBC-aware comparative structural profiles; exploratory multivariate profile analysis with robustness tests; PBC-aware lipid-specific contact-enrichment and persistence analysis; the controlled resistance decomposition; and PLU structural-contrast analysis (PDF)
