# Supplementary material for: Free-Energy and Position-Dependent Diffusion Profiles Shape Passive Permeation of a Semisynthetic Naphthoquinone across a Model Lipid Bilayer
Source: J Phys Chem B. 2026 Sep 4;130(37):9471–80. doi: 10.1021/acs.jpcb.6c02574 (PMC13592021; doi:10.1021/acs.jpcb.6c02574)

Supporting Information

Free-Energy and Position-Dependent Diffusion Profiles Shape Passive Permeation of a Semisynthetic Naphthoquinone across a Model Lipid Bilayer

Fábio J. N. Ferreira[1] *

Tallita Marques Machado[1]

Fernanda Guilhon-Simplicio[1,2]

[1] Faculty of Pharmaceutical Sciences, Federal University of Amazonas, Manaus, Amazonas, 69080-900, Brazil

[2] Center for Innovation in Artificial Intelligence for Health (CIIA – Saúde/UFMG), 31270-901, Brazil

* Corresponding Author E-mail: fabio.ferreira@ufam.edu.br

Figure S1. Steered Molecular Dynamics (SMD) Pulling Trajectory for CNFD. Time evolution of the center-of-mass z-coordinate during the 4.5 ns continuous pulling phase (nominal rate = 0.001 nm ps^{-1}, k = 1000 kJ mol^{-1} nm^{-2}). The plotted coordinate runs from z = -4.82 nm to approximately 0 nm. SMD was used only to generate candidate starting configurations; 46 configurations at approximately 0.1 nm spacing were subsequently equilibrated under umbrella restraints before ST-eUS production.

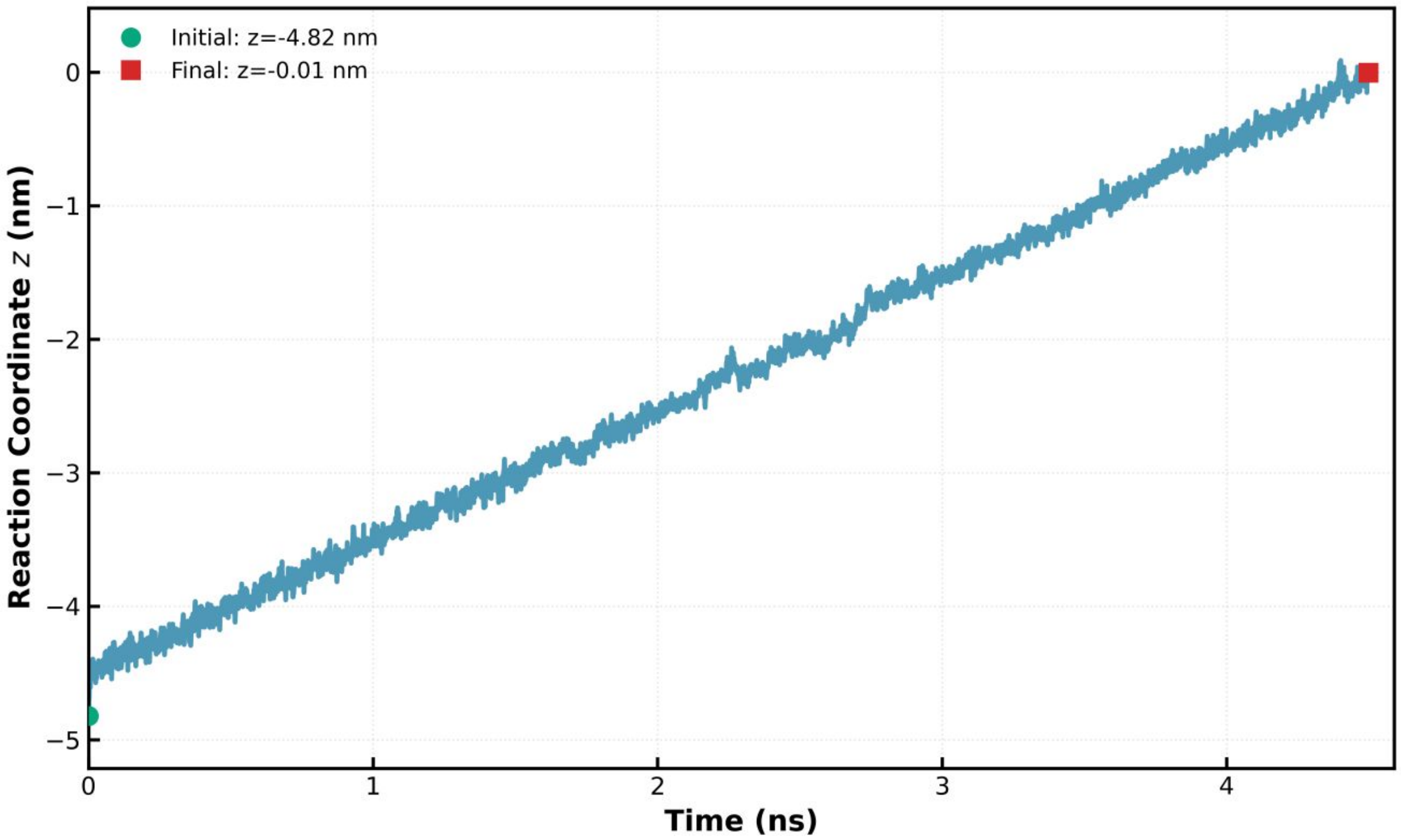

Figure S2. Convergence Analysis of ST-eUS. Overlay of PMF profiles from three independently seeded replicas (Replica 1, salmon; Replica 2, cyan; Replica 3, blue), each shifted by its own mean PMF at $z \geq 3.5$ nm. After interpolation onto the common coordinate interval, the three full-profile pairwise RMSDs were 1.260, 1.388, and 1.364 kJ mol^{-1}; their descriptive mean +/- sample SD was 1.34 ± 0.07 kJ mol^{-1}. Restriction to $z \leq 3.5$nm gave 1.51 ± 0.08 kJ mol^{-1}. These values quantify agreement among the three ST-eUS replicas and are not compared with a matched conventional-umbrella control.

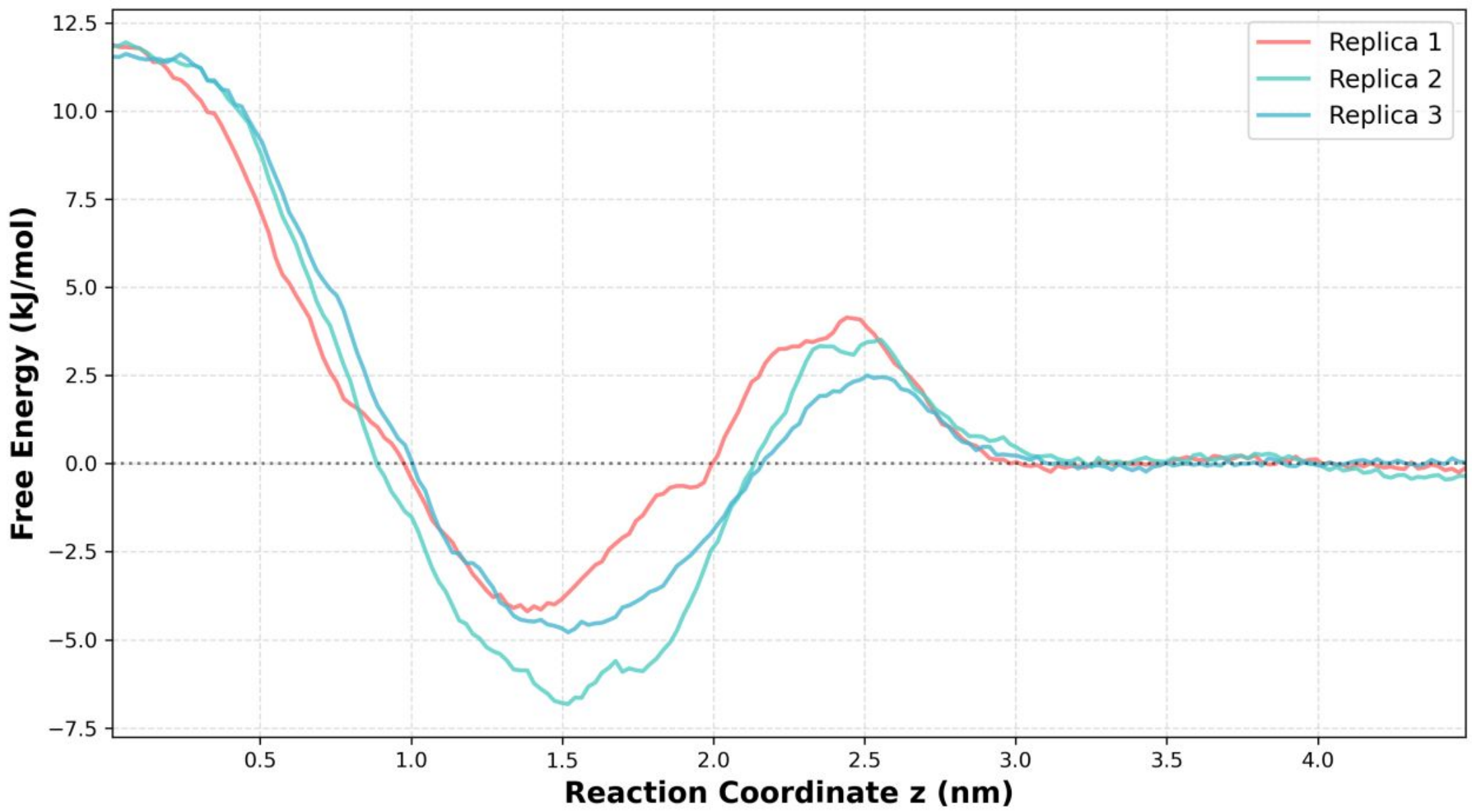

Figure S3. Primary Position-Dependent Diffusion Coefficient $D_{\perp}(z)$. The numerical-ACF/Hummer profile spans $D_{\mathrm{min}} = 2.79 \times 10^{-7}$ cm^2 s^{-1} at $z = 2.20$ nm to $D_{\mathrm{max}} = 1.62 \times 10^{-5}$ cm^2 s^{-1} at $z = 4.41$ nm, corresponding to $D_{\mathrm{max}}/D_{\mathrm{min}} = 57.9$. The fitted-PACF sensitivity profile instead spans 4.51×10^{-7} to 1.80×10^{-5} cm^2 s^{-1} (39.8-fold). The plotted line and band correspond to the primary numerical-ACF profile. The experimental bulk-water guide is not used to rescale $D_{\perp}(z)$.

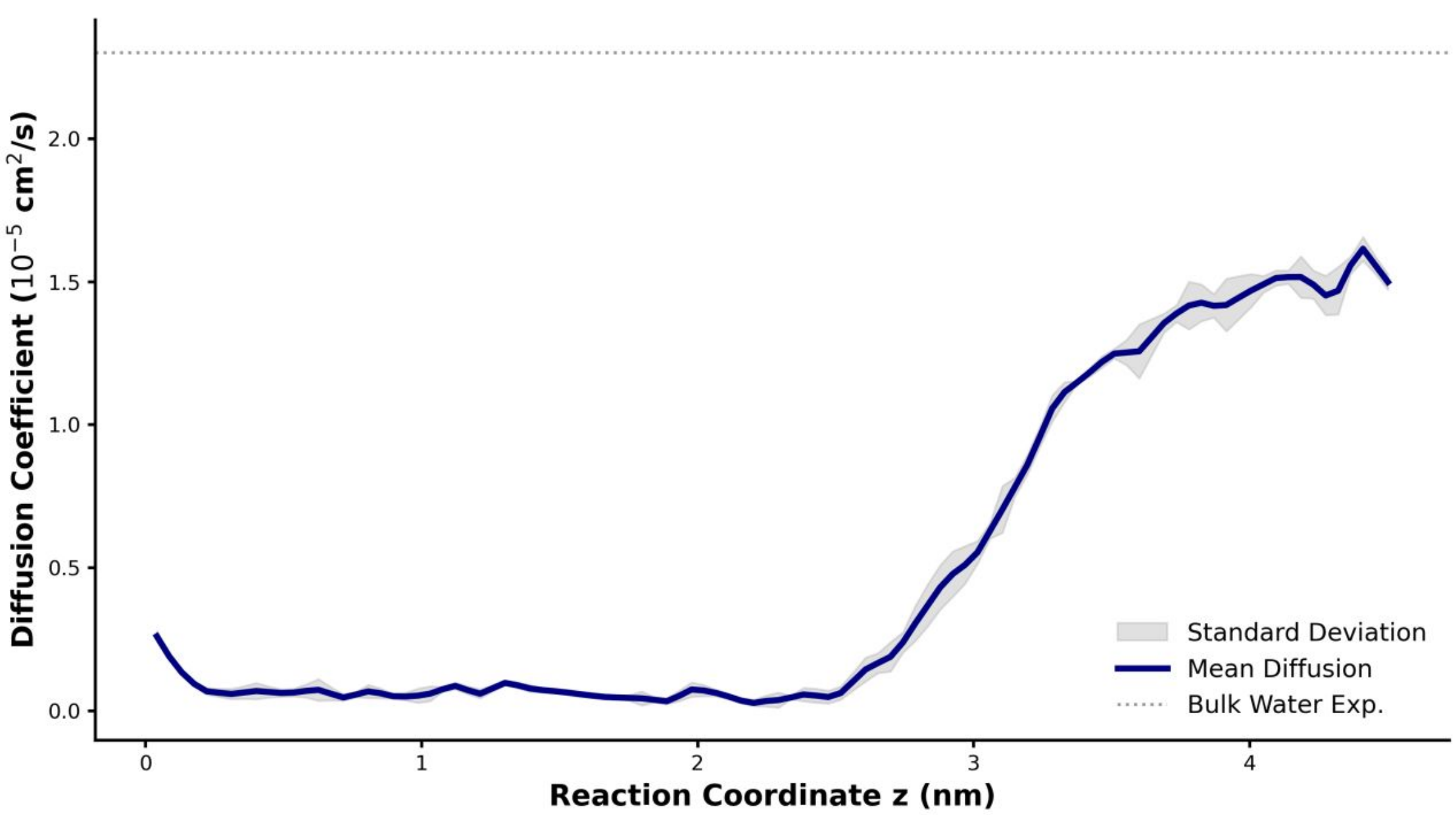

Figure S4. Bootstrap Error Analysis of the PMF Profile. Overlay of 50 independent bootstrap resampling profiles (light gray lines) with the resulting 95% confidence interval envelope (red shaded region) and the mean PMF trace (solid black line). The maximum half-width of the confidence interval is 1.60 kJ/mol across the sampled profile, confirming tight statistical convergence and high-confidence sampling of the free energy landscape.

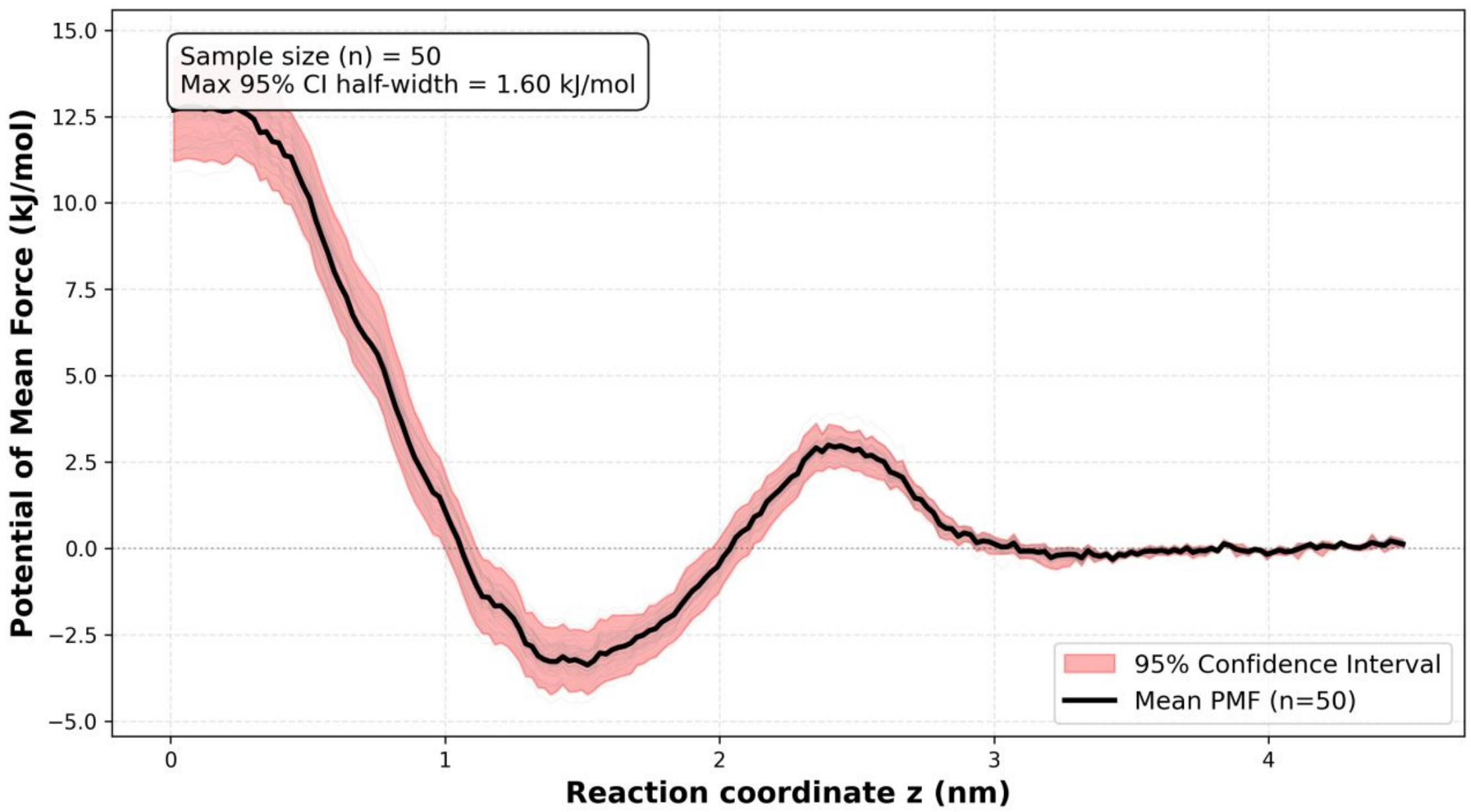

Figure S5. Sensitivity of CNFD intrinsic permeability to correlation-time treatment and representative PACF fit diagnostics. (A) Full-bilayer intrinsic P_{eff} obtained using numerical integration of the normalized positional autocorrelation function (primary analysis, 9.09×10^{-2} cm s[-1]) and parametric PACF fitting (sensitivity analysis, 1.14×10^{-1} cm s[-1]). Both estimates use the same Hummer positional-autocorrelation formalism. (B) Normalized PACF and bi-exponential fit for a representative trajectory near the headgroup-core transition (replica 1, window 22, $\langle z \rangle = 2.297$ nm). The fit over $0 < t \leq 200$ ps gave $A = 0.686$, $\tau_1 = 4.43$ ps, $\tau_2 = 202.13$ ps, $\tau_{\mathrm{corr}} = 66.51$ ps, and $R^2 = 0.922$. (C) Residuals, defined as PACF data minus fitted values. The representative was selected deterministically as the bi-exponential fit whose R^2 was closest to the median among trajectories with $2.1 \leq z \leq 2.4$ nm; it was not selected as a best-case fit.

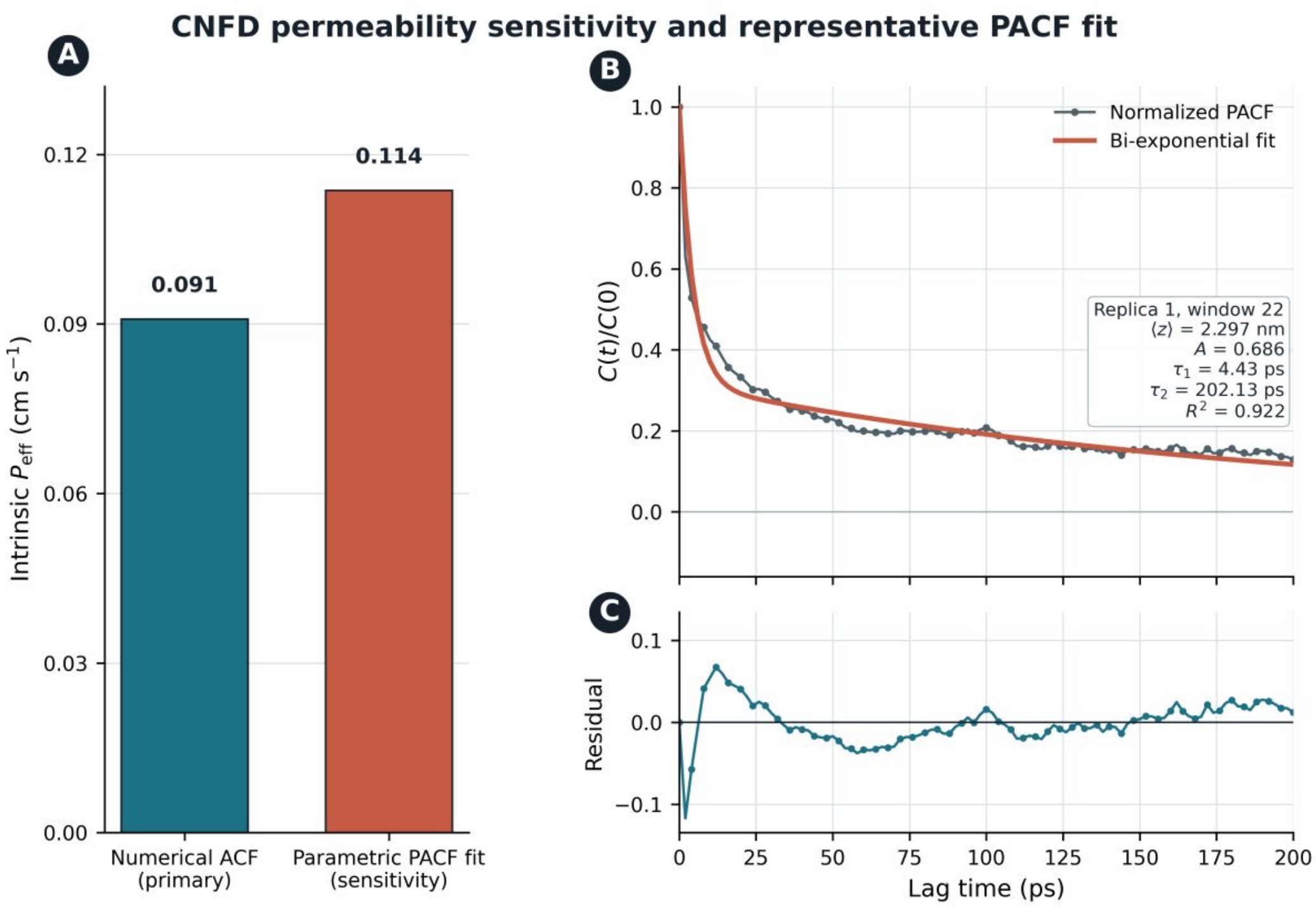

Figure S6. PBC-aware structural profiles for CNFD, beta-lapachone, and plumbagin. Water oxygens near ligand oxygens, nearby lipid residues, and ligand orientation are reported as mean +/- SEM across three replica-level window means. The coordinate is the absolute membrane distance reconstructed using the pull-reference atom under periodic boundary conditions. These profiles are mechanistic descriptors and not direct permeability estimates.

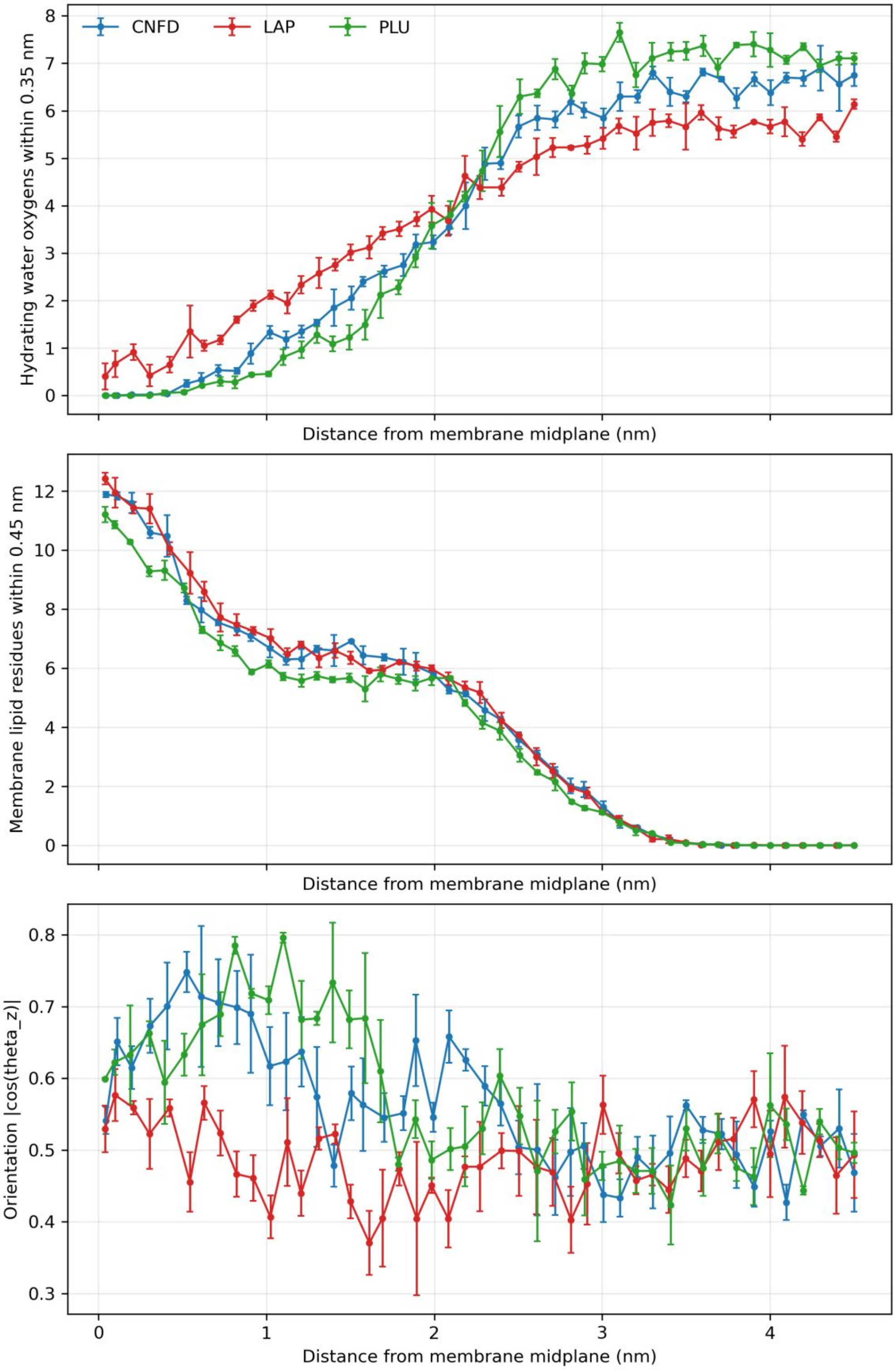

Exploratory Multivariate Profile Analysis

To examine whether the energetic, diffusional, hydration, contact, and orientation profiles contained recurring multivariate regimes, we performed an exploratory unsupervised analysis across the 46 ordered umbrella windows of CNFD, beta-lapachone, and plumbagin. The analysis used one compound-window profile per coordinate (138 rows) and retained replica variation only as a structural uncertainty layer; the rows were not treated as independent molecules. The primary F1 representation contained bulk-normalized PMF, $\log_{10}$ numerical-ACF/Hummer diffusion, PBC-aware hydration, and ligand orientation. The F2 sensitivity representation replaced PMF and diffusion with log10 local ISD resistance. The membrane coordinate z ordered and interpreted the profiles but was excluded from clustering, and integrated P_{eff} was never used as a feature.

Ward clustering of robust-scaled F1 profiles was compared with Gaussian mixtures, HDBSCAN, multivariate change-point detection, feature substitutions, alternative scaling, Hummer/PACF diffusion estimates, endpoint exclusion, and compound/replica omission tests. The primary Ward solution selected three descriptive clusters (silhouette 0.468; median block-bootstrap ARI 0.780; median consensus fraction 0.920). Consensus with ordered change points yielded four provisional segments for CNFD, four for beta-lapachone, and two for plumbagin.

CNFD and beta-lapachone reached central segments with mean PMF values of 11.65 and 12.73 kJ mol^{-1} and mean $\log_{10}$ local-resistance values of 7.93 and 7.84, whereas the central plumbagin segment retained a favorable mean PMF of -6.90 kJ mol^{-1} and mean $\log_{10}$ local resistance of 4.83. These profile-level contrasts were accompanied by progressive dehydration and increasing lipid contacts toward the membrane center.

The segmentation was not invariant. Resistance-based feature sets produced ARI values of 0.468-0.489 relative to the primary F1 partition, StandardScaler yielded ARI 0.634, and CNFD was sensitive to selected omission tests. Seven of nine Hummer/PACF boundary comparisons were within 0.20 nm; the plumbagin candidates at 1.73 and 0.67 nm were sensitive. Accordingly, the segments are descriptive and provisional. This analysis is exploratory, not predictive, not causal, and does not establish a transferable permeability model.

Figure S7. Integrated exploratory mechanistic comparison of CNFD, beta-lapachone, and plumbagin. (A) Harmonized bulk-normalized PMF profiles. (B) PBC-aware hydration (solid lines) and lipid-contact profiles (dashed lines). (C) Robust-scaled centroids of the provisional ordered segments. (D) Tested partition sensitivities, with the dashed vertical line indicating the ARI threshold of 0.65. The panel summarizes 138 ordered compound-window profiles from three molecular systems, not 138 independent molecules.

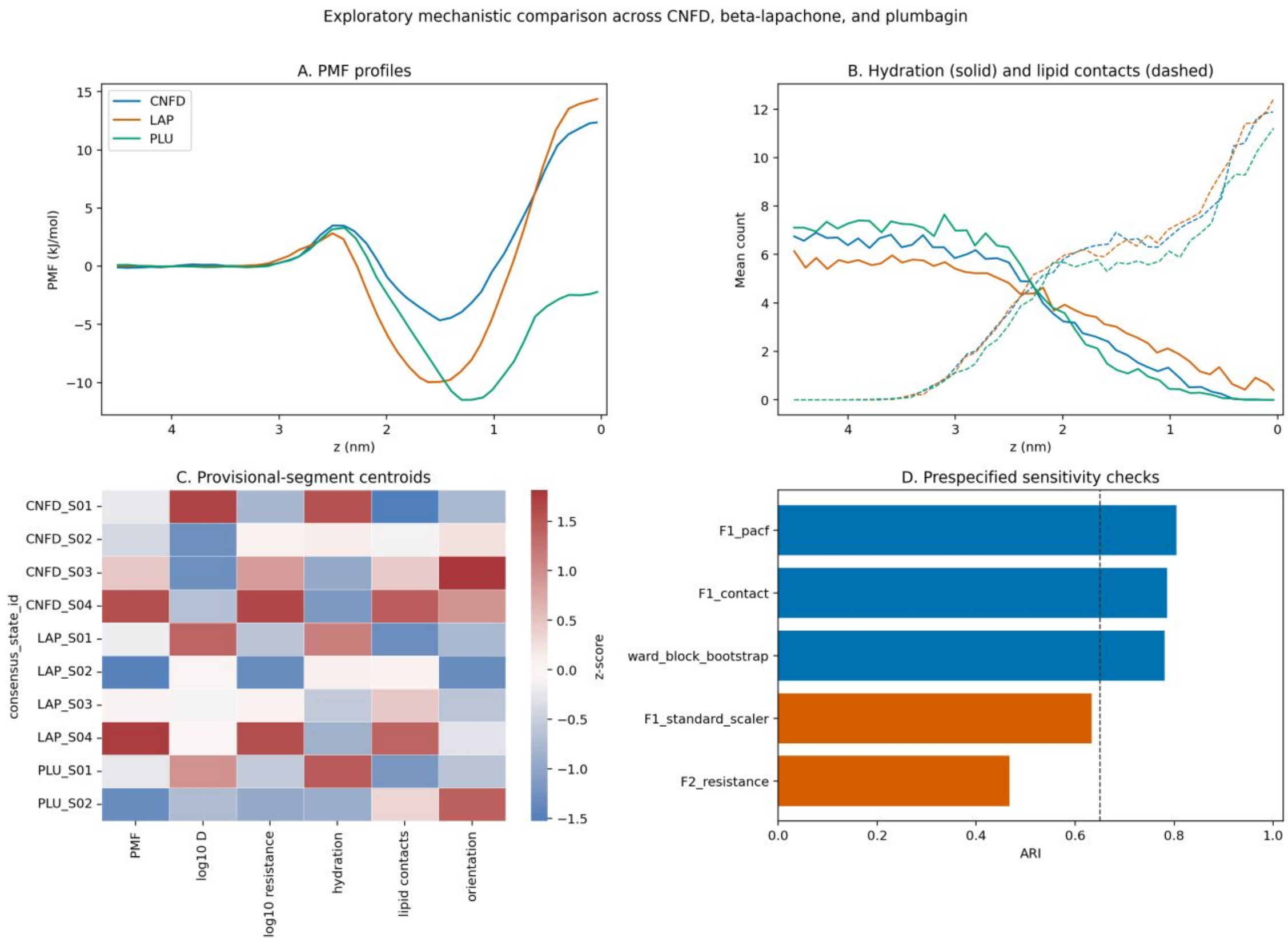

Figure S8. Robustness and ambiguity of the exploratory segmentation. The left panel reports adjusted Rand indices relative to the primary F1 Ward partition for feature, scaling, endpoint, compound-omission, and replica-omission sensitivities. The right panel reports displacement of Hummer-based change-point candidates when fitted-PACF diffusion replaces numerical-ACF/Hummer diffusion at fixed segmentation penalty. Blue results pass the defined threshold; orange results are retained as method sensitivity. The analysis does not constitute experimental validation or prediction for new molecules.

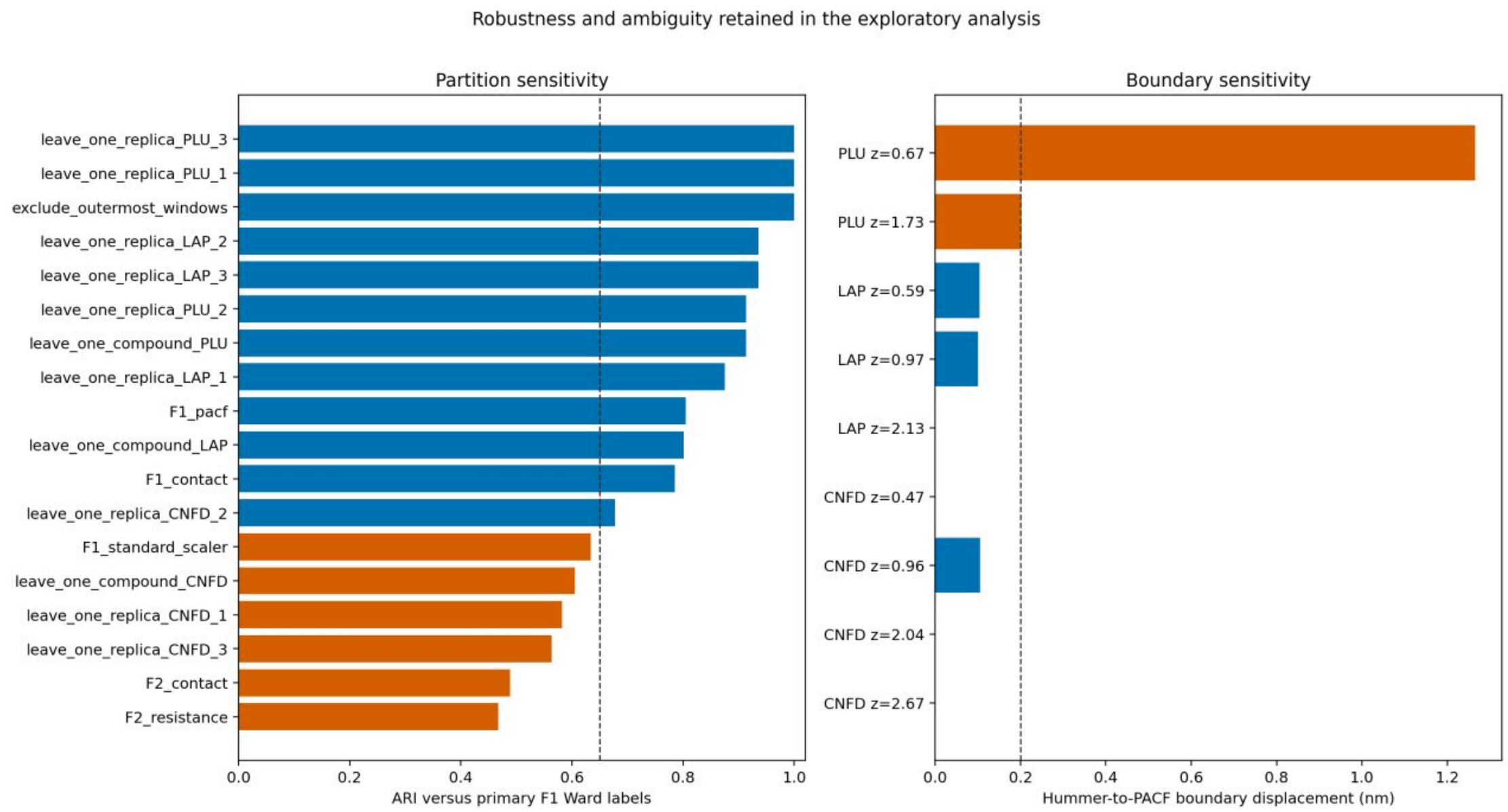

Figure S9. Lipid-specific contacts of CNFD along the membrane-normal coordinate. (A) Enrichment of POPC, POPE, cholesterol (CHL1), and PSM contacts relative to their local availability; lines and shaded regions show the mean and SEM across three replicas for estimable windows. The dashed line denotes enrichment equal to unity. (B) Fraction of analyzed frames containing at least one contact with each lipid species. (C) Median contact duration in seven representative windows, calculated at 2 ps temporal resolution with 0.45/0.50 nm formation/termination thresholds. Local enrichment features were not preserved across the full cutoff-sensitivity grid and therefore did not meet the defined criteria for lipid-specific association.

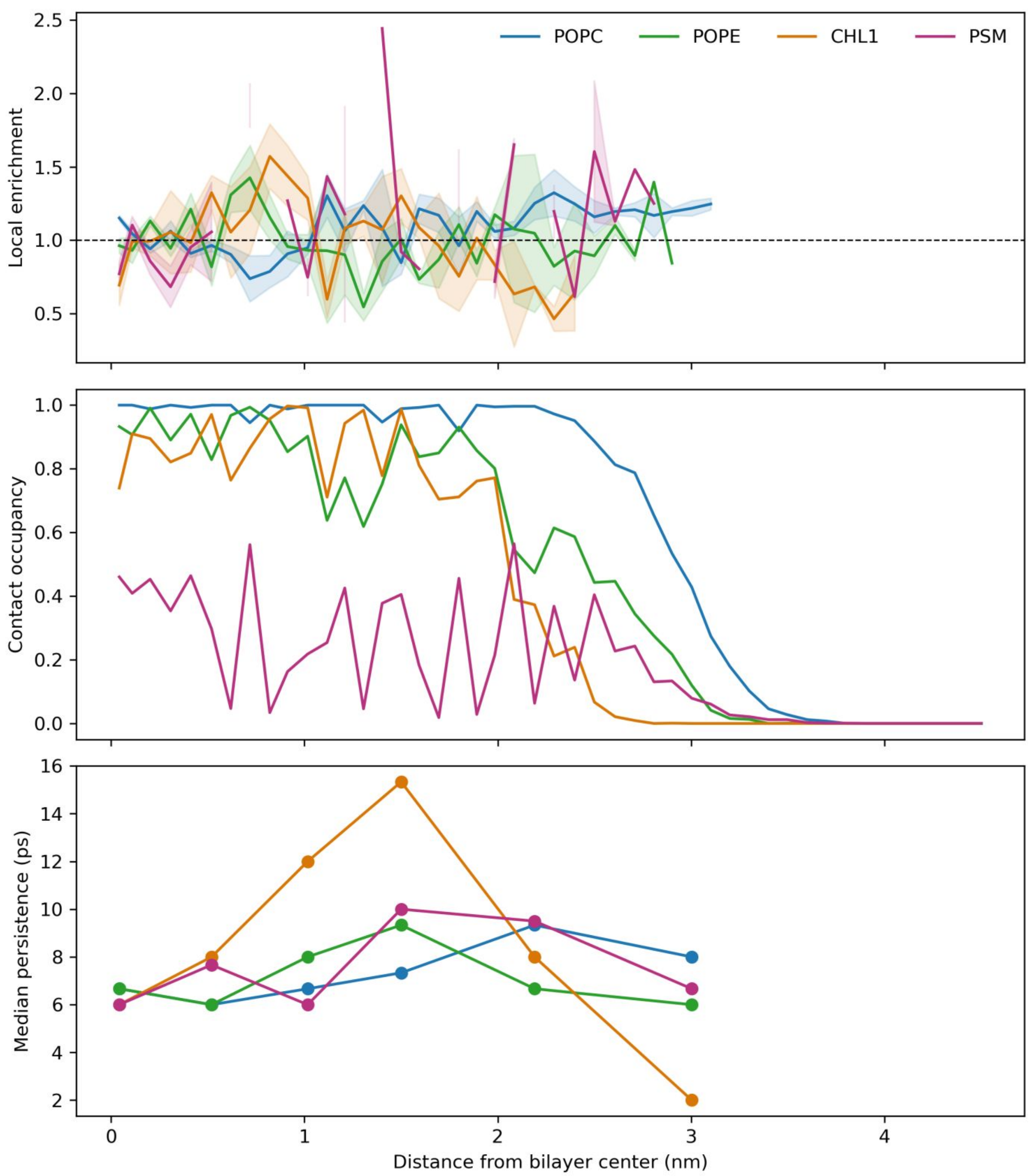

Controlled Resistance Decomposition and PLU Structural Contrast

Controlled substitutions were applied to the frozen PMF and diffusion profiles over the same half-bilayer interval. R_F retained the PMF with constant D_{bulk}; R_D retained $D_{\perp}(z)$ with a flat PMF; and R_{flat} used a flat PMF and D_{bulk}. The spatial mean at z ≥ 3.5 nm defined D_{bulk}. The exact factorization $R_{obs}/R_{flat} = A_F A_D \Gamma$ separates PMF amplification, diffusion amplification, and residual spatial coupling. These analytical counterfactuals were derived directly from the sampled profiles.

For CNFD, numerical-ACF and fitted-PACF profiles gave closely aligned conclusions: A_F = 10.54 versus 10.98, A_D = 14.89 versus 12.06, Γ = 1.134 versus 1.149, and center-resistance fractions of 81.4% versus 82.8%. Grid refinement changed permeability or Γ by at most 0.0415% across the tested resolutions. The choice of bulk-diffusion reference changes the numerical value assigned to A_D while leaving the observed permeability, Γ, resistance localization, or the qualitative compound contrast.

Table S1. Controlled resistance factors from the primary numerical-ACF profiles and the CNFD fitted-PACF sensitivity profile.

**Compound**	**Estimator**	A_F	A_D	**Γ**	**Center resistance (%)**	P_{eff} **(cm s−1)**
CNFD	numerical ACF	10.54	14.89	1.134	81.4	9.09 × 10−2
CNFD	fitted PACF	10.98	12.06	1.149	82.8	1.14 × 10−1
LAP	numerical ACF	21.19	4.85	1.453	94.0	1.06 × 10−1
PLU	numerical ACF	0.845	9.07	0.800	6.5	2.35

Figure S10. Near-center structural contrast among CNFD, β-lapachone (LAP), and plumbagin (PLU) for $z \leq 0.5$ nm. Bars show mean ± SEM across three replica-level summaries for local hydrating waters, neighboring lipid residues, lipid contacts normalized by ligand heavy-atom count, and principal-axis orientation relative to the membrane normal. PLU is smaller and essentially planar, is nearly dehydrated near the center, and has the largest contact count per heavy atom. A short O–H···O geometry was observed in the optimized PLU structure and three archived pulling-path configurations; these observations support a structural interpretation but do not establish equilibrium intramolecular-hydrogen-bond occupancy or PMF causality.

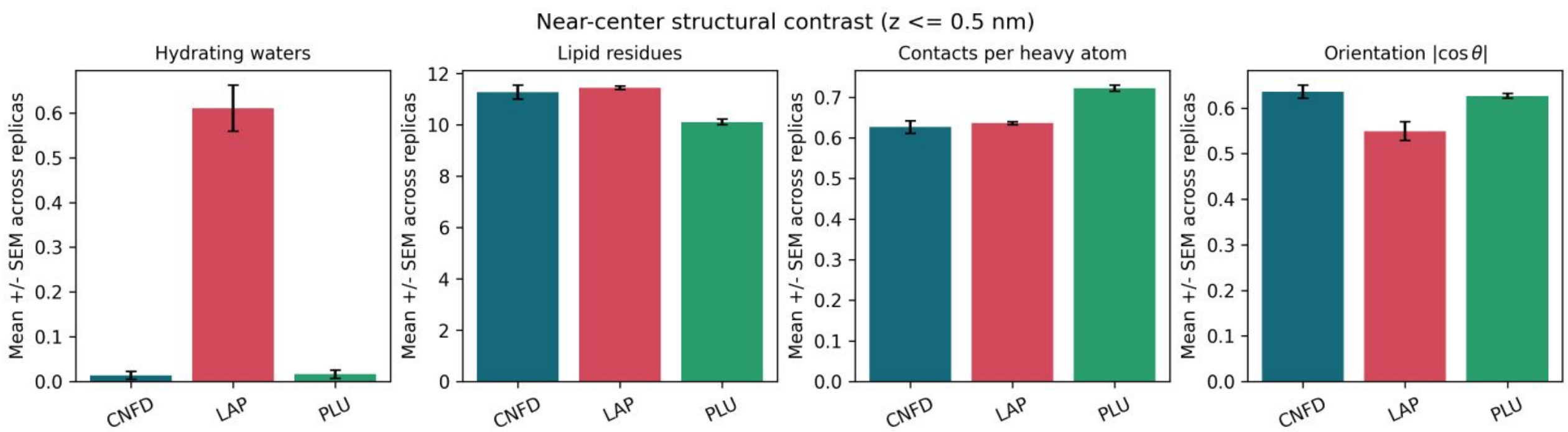

Supplement: Supplementary file 1 [file jp6c02574_si_001.pdf]
